# Modulation of autophagy and apoptosis can contribute to the anticancer effect of Abemaciclib/Celecoxib combination in colon cancer cells

**DOI:** 10.1007/s12032-023-02288-z

**Published:** 2024-01-03

**Authors:** Dalia Mohamed Elsayed Alian, Maged W. Helmy, Medhat Haroun, Nermine Moussa

**Affiliations:** 1 Department of Biotechnology, Institute of Graduate Studies and Research, Alexandria University, Alexandria, Egypt; 2 Department of Pharmacology and Toxicology, Faculty of Pharmacy, Damanhur University, Damanhur, Egypt

**Keywords:** Colon Cancer, Abemaciclib, Celecoxib, Autophagy, Apoptosis

## Abstract

Drug resistance and recurrence represent a great challenge in colorectal cancer management, highlighting the urgent need for novel therapeutics. Our objective is to evaluate the influence of Abemaciclib, Celecoxib, and their combination on both the autophagic and apoptotic machinery in an attempt to unravel the interplay between them in HCT-116 and Caco-2 cell lines. The MTT assay was used to assess the GI50 of the drugs. ELIZA was used to determine the protein levels of Beclin-1, LC3, Cox-2, and Bcl-2. Active Caspase-3 was determined by a colorimetric assay. Gene expression levels of ATG5, LC3, Beclin-1, and p62 were assessed by quantitative real-time PCR. In HCT-116 cells, the GI50s for Abemaciclib and Celecoxib were 15.86 and 92.67 μM, respectively, while for Caco-2 cells, the GI50s were 7.85 and 49.02 μM for Abemaciclib and Celecoxib, respectively. Upon treatment of HCT-116 and Caco-2 cells with Abemaciclib, Celecoxib, and their combinations, ATG5, p62, LC3, and Beclin-1 gene expression levels were up-regulated. The protein levels of Beclin-1, LC3, and Caspase-3 were significantly increased, while Bcl-2 was decreased in both cell lines due to single and combined treatments. Both drugs, either alone or in combination, decreased the migration ability of the cells in both cell lines. To conclude, the treatment protocol has the potential to induce cell cycle arrest, diminish the potentiality of cells for migration, and initiate apoptotic and autophagic cell death. Further research is recommended to unravel the potential antitumor effects of Abemaciclib/Celecoxib combination in different cancer types.

## Introduction

Colorectal cancer (CRC) is among the most frequent cancers globally and is the third leading cause of cancer-associated death. Although early identification and treatment have dramatically reduced CRC mortality, recurrence and drug resistance are major roadblocks. Accordingly, novel therapeutic modalities are urgently needed [1].

Autophagy and apoptosis are known to be deregulated in cancer, and their modulation holds great promise as a therapeutic strategy [2]. In response to various types of cellular stress, several levels of interaction exist between autophagy and apoptosis [3].

Autophagy plays a role in the transition from normal to cancerous colorectal cells, and its role in cancer seems to be context-dependent as it has both tumor suppressive and tumor-promoting roles [4]. Autophagy, a conserved catabolic process, involves cellular self-digestion and the elimination of damaged organelles and proteins. Initiation, elongation of the phagophore, maturation of autophagosomes and their fusion with lysosomes, and proteolytic degradation are the steps of autophagy. This process starts with the formation of the phagophore, which encloses the defective proteins. The phagophore is formed when several vesicles from the endoplasmic reticulum fuse together. The autophagosome’s outer membrane combines with the lysosome to produce the autolysosomes. The intra-lysosomal components are degraded by the lysosomal enzymes [5]. Various genes and proteins play crucial roles in this complicated autophagic pathway, including ATG5, p62, LC3, and Beclin-1.

Apoptosis is an essential biological process that involves the removal of damaged and excessive cells. It is also involved in several other biological processes, including ageing, tissue homeostasis, and cell growth. Incomplete apoptosis might result in cancer and autoimmune diseases. Numerous unique morphological changes in the cell’s structure, as well as a range of enzyme-dependent biological processes, are characteristics of apoptosis. Generally speaking, proteins including caspases, Bax, Bcl-2, p53, and cytochrome c are involved in the control of the apoptotic process. During the cell’s apoptotic process, pro-apoptotic proteins (Bax and Cyt C) and apoptosis inducing factor (AIF) levels rise simultaneously. On the other hand, apoptosis is inhibited by the anti-apoptotic protein Bcl-2, which does this by stopping the secretion of AIF and Cyt C along with caspase precursors [6–8].

Cyclin-dependent kinase 4/6 (CDK4/6)-cyclin D axis is deregulated in various cancer types. By collaborating with D-type cyclins and controlling the phosphorylation level of retinoblastoma (Rb), CDK4/6 facilitates the cell cycle transition from G1 to S phase. Un-phosphorylated Rb binds to and inhibits the activity of transcriptional factors from the E2 family (E2F). When E2F transcriptional factors are phosphorylated, Rb separates from them, allowing them to take part in DNA replication and cell division [9]. Abemaciclib (LY2835219), a selective CDK4/6 inhibitor, is active against an extensive range of solid tumors, including breast carcinoma, esophageal and non-small cell lung cancers, liposarcoma, and melanoma. Abemaciclib was approved by the FDA for breast cancer treatment and is still in clinical trials for other solid tumors [10]. Abemaciclib can inhibit Rb phosphorylation, thus stopping the cell cycle at the G1 phase. Abemaciclib arrests the cell cycle in CRC mouse xenografts. Also, it inhibited proliferation in mantle cell lymphoma, glioblastoma, and melanoma xenografts [9].

Cycloxygenase-2 (Cox-2) catalyzes the metabolic conversion of arachidonic acid to prostaglandins, which underlies Cox-2’s pro-inflammatory and tumor-promoting actions. Breast, ovarian, CRC, thyroid, and lung malignancies have all been reported to overexpress Cox-2. In precancerous and cancerous lesions of the colon, Cox-2 overexpression has been linked to reduced colon cancer cell apoptosis and increased angiogenesis-promoting factor production. Cox-2 levels are raised in colorectal adenomas and sporadic colon cancers, and Cox-2 overexpression in CRC is linked to a poorer prognosis. Inhibiting Cox-2 inhibits polyp development, restores apoptosis, and lowers the production of proangiogenic factors [11]. Celecoxib, a selective inhibitor of the Cox-2 enzyme, has potent anti-tumor activity in colorectal, breast, and lung cancers. Celecoxib can target a myriad of signal transduction pathways associated with Cox-2 expression in addition to its Cox-independent mechanisms [12].

To the best of our knowledge, the combination of Abemaciclib and Celecoxib hasn’t been tested before. In this context, our objective is to evaluate the influence of Abemaciclib as a selective CDK4/6 inhibitor and Celecoxib as a selective Cox-2 inhibitor, as well as their combination, on the autophagic machinery in HCT-116 and Caco-2 CRC cell lines via measuring the expression levels of LC3, Beclin-1, ATG5, and p62. Moreover, Caspase-3 and Bcl-2 are measured to unravel the interplay between autophagy and apoptosis in the investigated CRC cell lines.

## Materials & methods

### Materials

HCT-116 (ATCC® CCL-247™) and Caco-2 (ATCC® HTB-26™) cell lines were purchased from the American Type Culture Collection. Abemaciclib and Celecoxib were purchased from (Selleckchem, TX, USA). Dulbecco’s Modified Eagle’s Medium (DMEM), Penicillin/Streptomycin, and trypsin were obtained from Lonza Verviers SPRL, Belgium. Fetal bovine serum (FBS) and a human Caspase-3 assay kit were obtained from Sigma-Aldrich Co., Germany. T-25 flasks were purchased from Greiner Bio-One, Germany. Maxima SYBR Green/ROX qPCR Master Mix (2x) Thermo Scientific ™ and Thermo Scientific™ Pierce™ BCA kits were purchased from Thermo Fisher Scientific Inc., USA. All ELIZA kits were purchased from Abcam, USA. An Easy-spin ^TM^ total RNA extraction kit was purchased from Intron Biotechnology, South Korea. SensiFast™ cDNA synthesis kit was purchased from Bioline Co., USA.

#### Cell lines

HCT-116 is a CRC cell line obtained from an adult male, while Caco-2 is a human Caucasian colon adenocarcinoma obtained from the primary tumor of a 72-year-old Caucasian male utilizing the explant culture technique.

#### Drugs

Abemaciclib and Celecoxib were dissolved in deionized water and dimethyl sulphoxide (DMSO) at concentrations of 10 and 100 mM, respectively, then kept at -20 °C until the time of use.

### Methods

#### Cell culturing

Caco-2 and HCT-116 cells were maintained as a monolayer in T-25 flasks in DMEM (Phenol red, 4.5 g/l glucose, and L-glutamine) fortified with FBS (10% v/v) at 37 °C and 5% CO_2_. Penicillin (100 units/ml)/Streptomycin (100 μg/ml) were used.

#### Cell storage

Caco-2 and HCT-116 cells were aliquoted at 2 × 10^6^ viable cells/ml in a cryomedium (80% v/v DMEM, 10% v/v FBS, and 10% v/v DMSO). The cryovials were left at -80 °C overnight and then maintained in a liquid nitrogen tank until the time of use.

#### Cell thawing

Aliquots of complete growth medium (90% DMEM and 10% v/v FBS) were placed in a warm water bath. The cryovials were thawed, and the contents were transferred to a 15 ml falcon tubes containing complete medium (9 ml). Cells were re-suspended, then centrifugation was carried out at 12,000 rpm at 4°C for 10 min. Cells were dispersed in the complete medium (5 ml) and placed in T-25 flasks at a seeding density of about 4 × 10^4^ viable cells/cm^2^. After that, the flasks were kept at 37 °C in 5% CO_2_ to allow for cell attachment.

#### Sub-culturing of cells

When they became 80% confluent, Caco-2 and HCT-116 cells were passaged. The medium was removed by aspiration, and phosphate-buffered saline pH 7.2 (5 ml) was added to wash the medium from the adherent cells.

For detaching the adherent cells, trypsin (1 ml) (2.5% w/v) was added, and cells were kept for 5 min at 37^o^C. The flasks were tapped gently to detach cells, and cells were then seen using the inverted microscope (Micro Master inverted digital microscope, Thermo Fisher Scientific Inc., USA). After incubation, cells were detached, and the trypsin cell suspension was neutralized by adding an equal volume of the complete growth medium to the flasks. The cells were then dispersed over the monolayer surface by pipetting gently. The suspension was placed in tubes containing 5 ml of complete growth medium. After that, centrifugation was carried out at 4^o^C for 5 min at 12,000 rpm. The cells were then re-suspended in complete medium, transferred into new flasks at a seeding density of about 4 × 10^4^ viable cells/cm^2^, and kept at 37^o^C in 5% CO_2_.

#### Cell counting

For the determination of an inoculum with a suitable concentration for seeding, the haemocytometer was used for counting the cells. Briefly, the cell suspension was mixed with trypan blue and loaded into both chambers. Viable cells were counted via the inverted microscope at 10x magnification. The total number of cells can be known from the following equation:

$$\begin{array}{l}{\bf{Cells}}/{\bf{suspension}}\,{\rm{ = }}\,{\rm{Total}}\,{\rm{cells}}\,{\rm{counted}}\,{\rm{in}}\,{\rm{the}}\,{\rm{haemocytometer}}\,\\\,\,\,\,{\rm{sets}}\,{\rm{of}}\,{\rm{squares/4}}\,{\rm{ \times 1}}{{\rm{0}}^{\rm{4}}}{\rm{ \times }}\,{\rm{dilution}}\,{\rm{factor}}\,{\rm{ \times }}\,{\rm{volume}}\,{\rm{of}}\,{\rm{cell}}\,{\rm{suspension}}{\rm{.}}\end{array}$$

#### In-vitro cytotoxicity assay

Cytotoxicity was evaluated by the classic Microculture Tetrazolium Test (MTT), which reflects cell proliferation. MTT relies on the principle that tetrazolium (yellow) is reduced, giving formazan crystals (purple), which have maximum absorption at 540 nm [13].

In brief, HCT-116 and Caco-2 cells were plated in 200 μl DMEM containing Streptomycin (1 μg/ml), Penicillin (1 U/ml), and FBS (10% v/v). Then incubation was carried out at 37^o^C in 90% air and 10% CO_2_. The microtiter plate was then kept at 37^o^C in 5% CO_2_ for 24 h to allow for cell attachment. The concentrations tested for Abemaciclib were 34.88 μM, 17.44 μM, 8.72 μM, 4.36 μM, 2.18 μM, and 1.09 μM, while those for Celecoxib were 506.72 μM, 253.36 μM, 126.68 μM, 63.34 μM, 31.67 μM, and 15.84 μM. The combined concentrations tested for Abemaciclib and Celecoxib, respectively, were (34.88 μM-506.72 μM), (17.44 μM-253.36 μM), (8.72 μM-126.68), (4.36 μM-63.34 μM), (2.18 μM-31.67 μM), and (1.09 μM-15.84 μM).

The plate was then incubated for 48 h under the same conditions. On day three, the culture medium was discarded and the MTT reagent (10 μl) was added, followed by incubation for 4 h at the same conditions. After that, the supernatant was carefully removed so as not to disturb the formazan crystals. DMSO (100 μl) was added to solubilize the formazan crystals. The plate was left for 2 h in the dark. The optical density (OD) of each well was assessed at 570 nm using a microplate reader (Model 550, Bio-Rad, USA). The relation between the different concentrations of the drugs and percentage cell viability was plotted as a dose-effect curve, and the data was analyzed using CompuSyn 3.0.1 software.

#### Determination of the combination index

The combination index (CI) was determined to know if there is synergism, antagonism, or additive effect between Abemaciclib and Celecoxib, where values less than 1 indicate synergism, values equal to 1 indicate additive effect, and values greater than 1 indicate antagonism.

#### Treatment of HCT-116 and Caco-2 cells with the selected drugs

Caco-2 and HCT-116 cells were divided into 5 groups including: (a) Control cells (untreated cells) in complete growth medium as a vehicle; (b) Abemaciclib-treated cells: Abemaciclib was dissolved in deionized water and diluted to a final concentration of 15.86 μM for HCT − 116 cells and 7.85 μM for Caco-2 cells with the complete medium; (c) Celecoxib-treated cells: Celecoxib was dissolved in 1% DMSO and diluted to a final concentration of 92.67 μM for HCT-116 cells and 49.02 μM for Caco-2 cells with the complete medium; (d) Abemaciclib/Celecoxib-treated cells (combination 1) (at their growth inhibition 50 (GI50)): Abemaciclib and Celecoxib were dissolved in 1% DMSO and diluted to a final concentration of 15.86 μM and 92.67 μM, respectively for HCT-116 cells and 7.85 μM and 49.02 μM, respectively for Caco-2 cells; and (e) Abemaciclib/Celecoxib-treated cells (combination 2) (at reduced GI50 based on the calculated dose reduction index): Abemaciclib and Celecoxib were dissolved in 1% DMSO and diluted with complete medium to a final concentration of 3.45 μM and 50.08 μM, respectively, for HCT-116 and 2.26 μM and 32.76 μM, respectively, for Caco-2 cells. On day 3, cell pellets were collected and kept at -80^o^C until the time of use.

### Analysis of ***ATG5***, ***LC3***, ***Beclin-1***, and P62 gene expression by quantitative real time PCR

#### Total RNA isolation

To successfully isolate total RNA, an Easy-spin ^TM^ total RNA extraction kit was used according to the manufacturer’s instructions. Briefly, Easy-blue lysis buffer (1 ml) was added to the cell pellet after centrifugation. Then, the samples were vortexed for 10 s. Chloroform (200 μl) was added. Samples were centrifuged at 13,000 rpm at 4 °C for 10 min. Then, the upper fluid (400 μl) was placed in eppendorf tubes, followed by the addition of 400 μl of the binding buffer. Samples were loaded into the column, followed by centrifugation at 13,000 rpm for 30 s. After that, the flow-through was discarded, and the spin column was placed back in the 2 ml collection tube. Then, washing buffer A (700 μl) was added, followed by centrifugation at 13,000 rpm for 30 s. After discarding the flow-through, washing buffer B (700 μl) was added, followed by centrifugation at 13,000 rpm for 1 min. The spin column was centrifuged at 13,000 rpm for 2 min to dry the column membrane. Finally, the column was placed in a clean tube, and the elution buffer (50 μl) was placed on the membrane. Incubation was carried out for 1 min, followed by centrifugation at 13,000 rpm for 60 s.

#### Total RNA quantification and purity checking

The concentration and purity of the extracted total RNA were assessed using a Nano Drop 2000 spectrophotometer (Thermo Fischer Scientific, USA). The OD was measured at 260, 280, and 230 nm using Tris-EDTA buffer as a blank. The concentration of total RNA was assessed by the OD reading at 260 nm as per the following conversion: an A260 of 1.0 is equivalent to 40 μg/ml of RNA. The A260/A280 ratio was determined as an indication of protein contamination, as the A260/A280 ratio for pure RNA is 2.1; however, values between 1.8 and 2.0 were considered acceptable. Also, A260/A230 ratio was determined as a measure of phenolic compound contamination, where an A260/A230 ratio greater than 1.5 was acceptable.

#### Complementary DNA synthesis

A complementary DNA (cDNA) synthesis step was performed utilizing SensiFast™ cDNA synthesis kit according to the manufacturer’s instructions to deliver highly robust first strand synthesis and higher cDNA yields.

#### Quantitative real time PCR

Quantitative real time PCR was done to determine the relative expression level of *ATG5*, *LC3*, *Beclin1*, and *p62* against a housekeeping gene (*β*-actin), depending on ∆∆CT method. A quantitative real time PCR assay was performed by the Rotor-Gene Q system (Qiagen, Germany). The Maxima SYBR Green/ROX qPCR Master Mix (2x) Thermo Scientific ™ kit was used according to the manufacturer’s instructions.

Table 1 presents the sequences of the primers used. To confirm the expected unique amplification of *ATG5*, *LC3*, *Beclin-1*, *p62*, and *β-actin*, the primer sequences were blasted against NCBI/Primer Blast. The analyses were done three times. Each sample gave an amplification curve, which is the PCR cycle number when a threshold fluorescence value is reached. The threshold value was confirmed to be within the exponential phase of the amplification curve. The comparative threshold (CT) value was utilized to calculate the normalized target. ΔCT values were determined by subtracting the CT value of the target gene (*ATG5*, *LC3*, *Beclin1*, and *P62*) from that of the reference gene (*β*-actin). ΔΔCT was determined from the equation: ΔΔCT = ΔCT_treated_ - ΔCT_control_, and the relative quantification (RQ) was determined as 2^−∆∆CT^. The % change in gene expression was calculated from the following Eq. (1-RQ) *100, and the fold changes were obtained by normalizing (2^−∆∆CT^) to the control value.

**Table 1:** Sequences of the primers

Genes	Forward &reverse primer sequences
*ATG5*	**Forward**: 5´-CAACTTGTTTCACGCTATATCAGG- 3´ **Reverse**: 5´- CACTTTGTCAGTTACCAACGTCA- 3´
*LC3*	**Forward**: 5’- GATGTCCGACTTATTCGAGAGC − 3’ **Reverse**: 5’- TTGAGCTGTAAGCGCCTTCTA − 3’
*Beclin-1*	**Forward**: 5’- GAGGGATGGAAGGGTCTAAG − 3’ **Reverse**: 5’- GCCTGGGCTGTGGTAAGT − 3’
*P62*	**Forward**: 5′-TGAGGAACAGATGGAGTCGG-3′ **Reverse**: 5′-GAGATGTGGGTACAAGGCAG-3′
*β- actin*	**Forward**: 5’-CTGGAACGGTGAAGGTGACA − 3’ **Reverse**: 5’-AAGGGACTTCCTGTAACAATGCA − 3’

#### Determination of the total protein content

To assess the total protein content in the cell lysates, the Thermo Scientific™ Pierce™ BCA protein assay was used as per the manufacturer’s instructions. Bovine serum albumin (standard) was dissolved in the cell lysis solution at concentrations of (125, 250, 500, 750, 1000, 2000, 3000, and 4000 μg/ml). The cell lysates were diluted by the lysis buffer 10 times. Duplicates of 10 ml aliquots of the standard solutions and diluted lysates were placed in appropriate tubes, and then the working reagent (200 μl) was added. All tubes were vortexed, left for 2 min, and then the OD was determined at 562 nm.

#### Biochemical analyses using the ELISA technique

The protein levels of Beclin-1, LC3B II, Bcl-2, and Cox-2 were determined using the appropriate ELIZA kits according to the manufacturer’s instructions.

#### Determination of active Caspase-3 in Caco-2 and HCT-116 cell lysates

To determine the active Caspase-3 protein level in Caco-2 and HCT-116 cell lysates, a human Caspase-3 assay kit was used, which relies on the ability of Caspase-3 to hydrolyze the peptide substrate (acetyl-Asp-Glu-Val-Asp p-nitroanilide), yielding the p-nitroaniline (pNA) moiety. The pNA concentration was known from the OD values at 405 nm. First, 10 μl of cell lysates or Caspase-3 positive controls were placed in the proper tubes, and the assay buffer (1x) was added as shown in the manufacturer’s instructions. Then, Caspase-3 inhibitor (10 μl) was used, and the reaction was initiated by adding Caspase-3 substrate (10 μl), followed by incubation for 2 h at 37 °C and the OD was determined at 405 nm. The results were calculated as μmol of pNA released per minute per ml of cell lysate or positive control based on the formula:

$${\rm{Activity}}\,\left( {{\rm{\mu mol}}\,{\rm{pNA/min/ml}}} \right)\, = \,\frac{{{\rm{OD}} \times {\rm{d}}}}{{\epsilon \times t \times v}}$$

Where: OD: observed optical density.

ε: pNA absorptivity in mM (10.5).

v: volume of sample in ml.

d: dilution factor.

t: reaction time in minutes.

#### Cell migration assay

Cell migration was performed using the *in-vitro* wound healing assay as described previously [14]. In 6-well plates, cells were seeded and left for 48 h to form a confluent monolayer, and the culture dishes were placed inside the incubator. A sterile plastic micropipette tip was used to simulate an *in-vivo* wound in every well. The pipette was angled appropriately, and constant pressure was applied to create a constant gap width. After making the scratch, the monolayer was washed with basal medium so as to remove the cell debris. In the case of HCT-116 cells, complete medium containing 3.17 μl, 1.85 μl, (3.17 μl/1.85 μl), (0.69 μl/1 μl) for Abemaciclib, Celecoxib, combination 1 and combination 2, respectively, was added, while for caco-2 cells, complete medium containing 1.569 μl, 0.98 μl, (1.569 μl/0.98 μl), (0.45 μl/0.655 μl) for Abemaciclib, Celecoxib, combination 1 and combination 2, respectively, were added to the wells. Experiments were done with cells in a tissue culture incubator kept at 37 ºC, 5% CO_2_, and 95% air. Migration progress was documented by taking photographs of the gap via the bright-field microscope. Finally, the data analysis was done according to the following equation:

$$\begin{array}{l}{\rm{Migratory}}\,{\rm{cells}}\,{\rm{\% }}\,{\rm{in}}\,{\rm{the}}\,{\rm{scratch}}\,{\rm{zone}}\,{\rm{ = }}\\\frac{{{\rm{No}}\,{\rm{of}}\,{\rm{cells}}\,{\rm{in}}\,{\rm{the}}\,{\rm{control}}\,{\rm{sample}}\,{\rm{ - }}\,{\rm{No}}\,{\rm{of}}\,{\rm{cells}}\,{\rm{in}}\,{\rm{the}}\,{\rm{treated}}\,{\rm{sample}}}}{{{\rm{No}}\,{\rm{of}}\,{\rm{cells}}\,{\rm{in}}\,{\rm{the}}\,{\rm{control}}\,{\rm{sample}}}}\end{array}$$

### Statistical analysis of the data

Data was shown as mean ± standard error of the mean. The obtained results were examined by means of one-way analysis of variance then by Tukey post hoc test. The analyses were done utilizing the Graph Pad Prism Software (version 6.0). The level of significance was set at *p* < 0.05.

## Results

### GI50 determination for Abemaciclib and Celecoxib in the investigated CRC cells

**Fig. 1:**
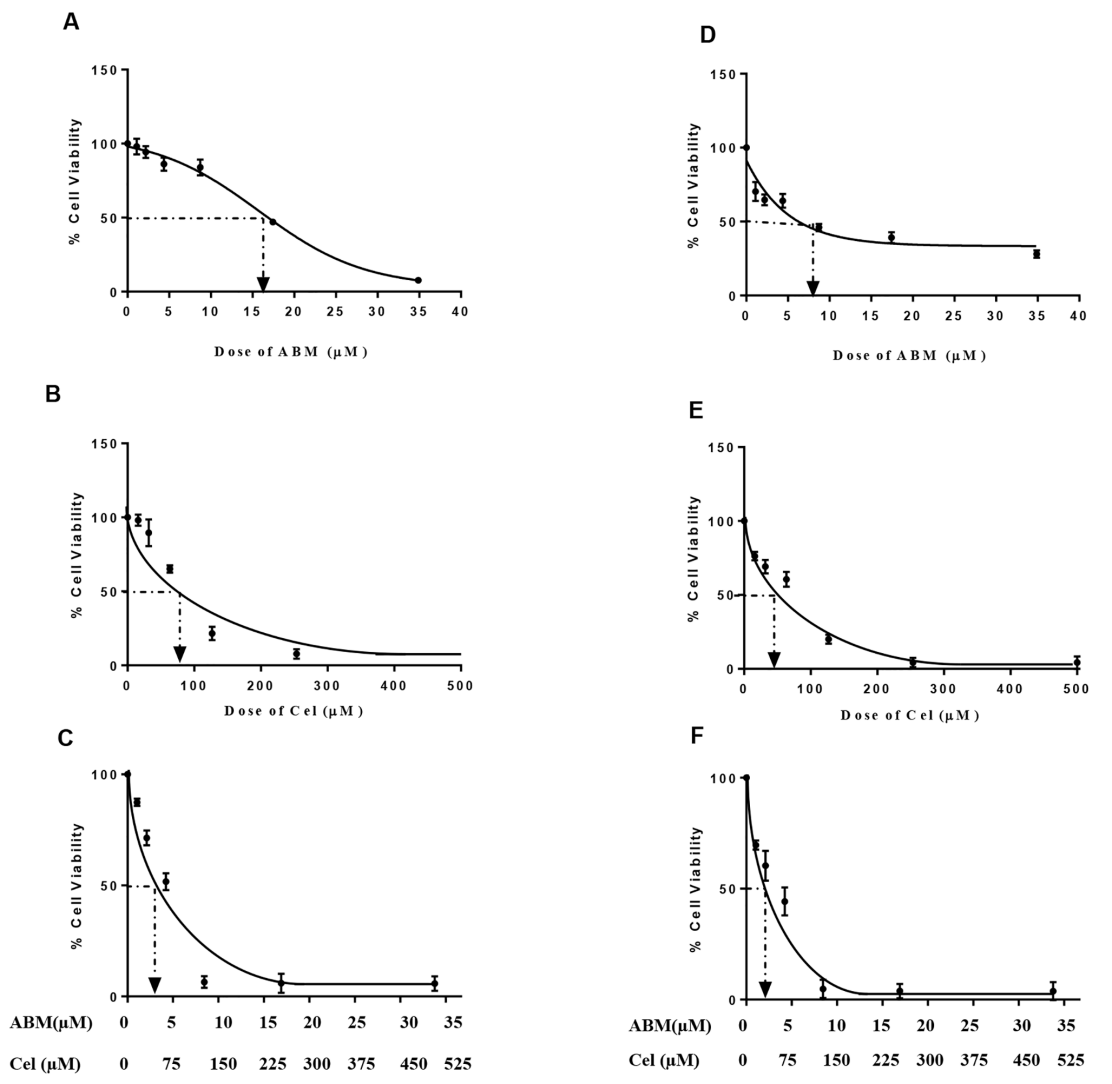
(**A**, **B**, **C**) Dose-response curve for Abemaciclib, Celecoxib and their combination in HCT-116 cells using MTT assay. (**D**, **E**, **F**) Dose-response curve for Abemaciclib, Celecoxib and their combination in Caco-2 cells using MTT assay

According to the MTT assay, the doses that induced 50% growth inhibition in HCT-116 cells were 15.86 μM and 92.67 μM for Abemaciclib and Celecoxib, respectively, as shown in (Fig. 1A and B), while such doses in Caco-2 cells were 7.85 μM and 49.02 μM for Abemaciclib and Celecoxib, respectively, as illustrated in (Fig. 1D and E).

### Determination of the combination and dose reduction indices

Based on the MTT assay results and the statistical analyses using Compusyn software, a synergistic effect was found between both drugs in HCT-116 and Caco-2 cells, as evidenced by the combination indices (CI = 0.7578, CI = 0.956, respectively). In HCT-116 cells, analysis of the dose reduction index (DRI) inferred that Celecoxib reduced the Abemaciclib dose by about 4.6 folds and Abemaciclib decreased the dose of Celecoxib by almost 1.85 folds, as shown in (Fig. 1C). Likewise, analysis of the DRI in Caco-2 cells showed that Celecoxib reduced the Abemaciclib dose by around 4.5 folds and Abemaciclib reduced the dose of Celecoxib by about 1.5 folds, as illustrated in (Fig. 1F).

### Effect of the treatment protocol on the autophagic markers (LC3, ATG5, P62, and Beclin-1) in the investigated CRC cells after 48 h of treatment

#### Influence of the treatment protocol on ***ATG5*** expression level in HCT-116 and Caco-2 cells after 48 h of treatment

**Fig. 2:**
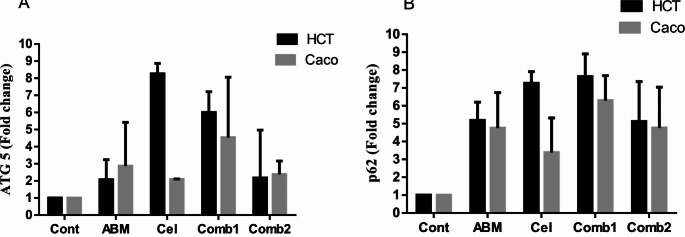
(**A**) Influence of the treatment protocol on *ATG5* gene expression level in HCT-116 and Caco-2 cells after 48 h of treatment. (**B**) Influence of the treatment protocol on *p62* gene expression level in HCT-116 and Caco-2 cells after 48 h of treatment

The data presented in (Fig. 2A) inferred that treatment of HCT-116 cells with Abemaciclib, Celecoxib, and their combinations increased the *ATG5* gene expression level in comparison to the control group by 2.1, 8.2, 6, and 2.2 folds, respectively (*p* < 0.8985, *p* < 0.001, *p* < 0.0140, and *p* < 0.8667, respectively). Pertaining to Caco-2 cells, both drugs and their combinations increased the *ATG5* gene expression level by 2.9, 2.1, 4.5, and 2.4 folds compared to the control group, respectively (*p* < 0.7364, *p* < 0.001, *p* < 0.5587 and *p* < 0.2590, respectively), as presented in (Fig. 2A).

#### Influence of the treatment protocol on ***p62*** gene expression level in the investigated CRC cells after 48 h of treatment

As shown in (Fig. 2B), treatment of HCT-116 cells with Abemaciclib, Celecoxib, and their combinations elevated the *p62* gene expression level by 5.2, 7.3, 7.6, and 5.1 folds, respectively (*p* < 0.0157, *p* < 0.0009, *p* < 0.0006, and *p* < 0.0171, respectively) when compared to the control group. The same pattern was seen in Caco-2 cells, where Abemaciclib, Celecoxib, and their combinations increased the *p62* gene expression level by 4.8, 3.4, 6.3, and 4.8 folds, respectively (*p* < 0.2348, *p* < 0.4373, *p* < 0.0672, and *p* < 0.2921, respectively) in comparison to the control group as given in (Fig. 2B).

#### Influence of the treatment protocol on ***Beclin-1*** gene and protein expression levels in the investigated CRC cells after 48 h of treatment

**Fig. 3:**
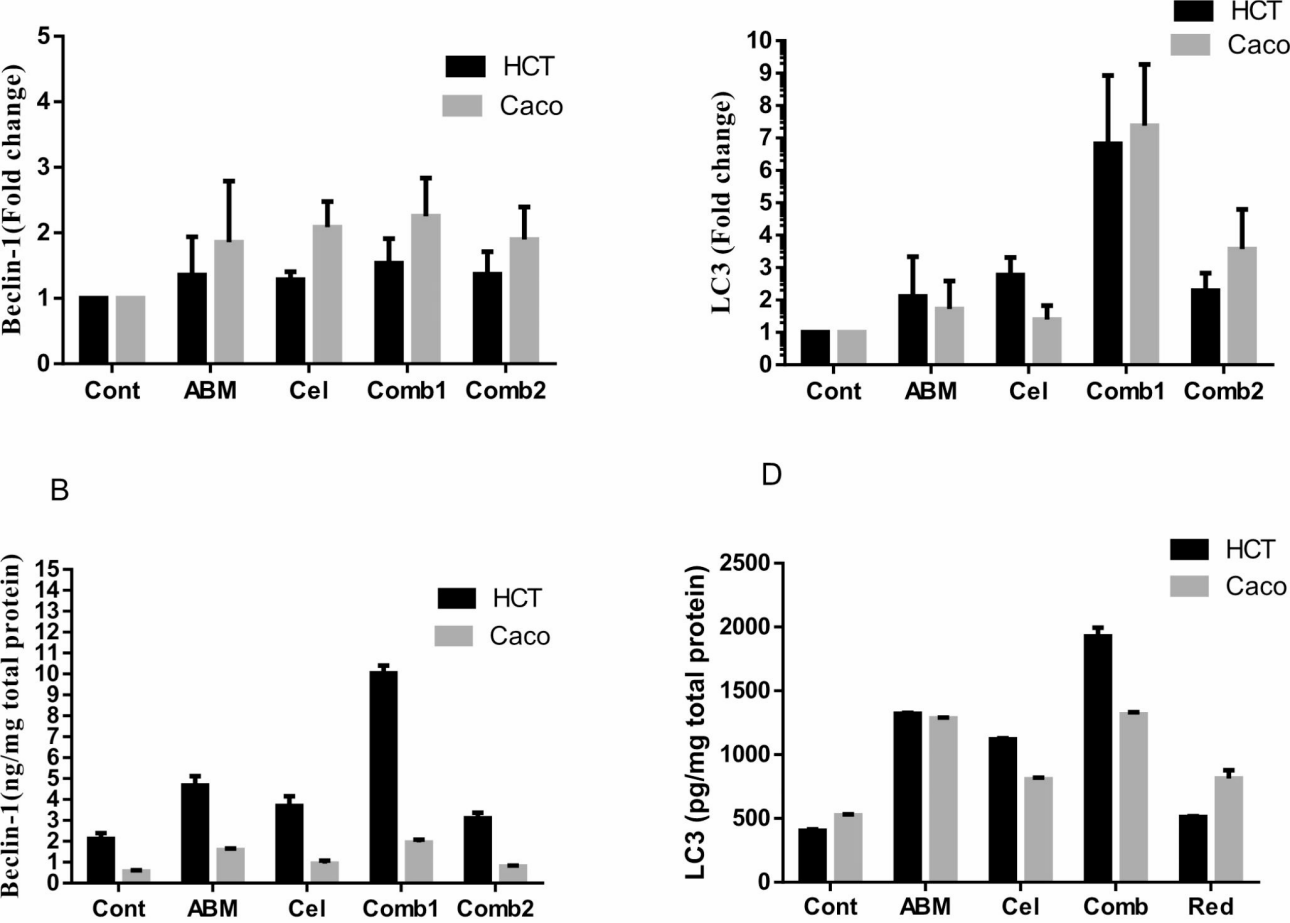
(**A**) Influence of the treatment protocol on *Beclin-1* gene expression level in HCT-116 and Caco-2 cells after 48 h of treatment. (**B**) Influence of the treatment protocol on Beclin-1 protein level in the investigated CRC cells after 48 h of treatment. a: *p* < 0.05 vs. control, b: *p* < 0.05 vs. Abemaciclib, c: *p* < 0.05 vs. combination 1, d: *p* < 0.05 vs. combination 2 where a, b, c, d denotes statistical significance of the treated groups versus each other as shown in the figure. (**C**) Influence of the treatment protocol on *LC3* gene expression level in HCT-116 and Caco-2 cells after 48 h of treatment. (**D**) Influence of the treatment protocol on LC3 protein level in the investigated CRC cells after 48 h of treatment. a: *p* < 0.05 vs. control, b: *p* < 0.05 vs. Abemaciclib, c: *p* < 0.05 vs. combination 1, d: *p* < 0.05 vs. combination 2 where a, b, c, d denotes statistical significance of the treated groups versus each other as shown in the figure

Upon treatment of HCT-116 cells with Abemaciclib, Celecoxib, and their combinations, the *Beclin-1* gene expression level was increased by 1.4, 1.3, 1.5, and 1.4 folds, respectively (*p* < 0.7271, *p* < 0.8491, *p* < 0.3933, and *p* < 0.7015, respectively) in comparison to the control group as presented in (Fig. 3A). Likewise, the data herein (Fig. 3A) showed that treatment of Caco-2 cells with Abemaciclib, Celecoxib, and their combinations elevated the *Beclin-1* gene expression level by 1.9, 2.1, 2.3, and 1.9 folds, respectively (*p* < 0.6106, *p* < 0.1191, *p* < 0.1896, and *p* < 0.2487, respectively) when compared to the control group.

The results shown in (Fig. 3B) revealed that the Beclin-1 protein level in HCT-116 cells was significantly increased by 120%, 75%, and 374% compared to the control in Abemaciclib-treated, Celecoxib-treated, and combination 1-treated cells, respectively (*p* < 0.0001, *p* < 0.0030, and *p* < 0.0001, respectively). Moreover, a statistically significant difference between Abemaciclib-treated, Celecoxib-treated, and combination 2-treated cells compared to the combination 1-treated cells (*p* < 0.0001) was found. The same pattern was seen in Caco-2 cells, where such levels were significantly elevated by 183%, 67%, and 246% compared to the control group in Abemaciclib-treated, Celecoxib-treated, and the combination 1-treated cells, respectively (*p* < 0.0001, *p* < 0.007 and *p* < 0.0001 respectively). Moreover, a statistically significant difference between Abemaciclib-treated, Celecoxib-treated, and combination 2-treated cells compared to the combination 1-treated cells (*p* < 0.0108, *p* < 0.0001, and *p* < 0.0001, respectively) was evident, as demonstrated herein (Fig. 3B).

#### Influence of the treatment protocol on ***LC3*** gene and protein expression levels in the investigated CRC cells after 48 h of treatment

The *LC3* gene expression level was found to be up-regulated upon treatment of HCT-116 cells with Abemaciclib, Celecoxib, and their combinations by 2.1, 2.8, 6.8, and 2.3 folds, respectively (*p* < 0.7585, *p* < 0.3775, *p* < 0.0007, and *p* < 0.6547, respectively) when compared to the control group, while such a level in Caco-2 cells was found to be up-regulated by 1.7, 1.4, 7.3, and 3.6 folds, respectively (*p* < 0.6620, *p* < 0.5978, *p* < 0.0844, and *p* < 0.1992, respectively) in comparison to the control group as presented in (Fig. 3C).

The data presented herein (Fig. 3D) showed that LC3 protein levels in HCT-116 cells were significantly increased by 228%, 179%, 379%, and 27% in relation to the control in Abemaciclib-treated, Celecoxib-treated, combination 1-treated, and combination 2-treated cells, respectively (*p* < 0.0001, *p* < 0.0001, *p* < 0.0001, and *p* < 0.0121, respectively). Moreover, there was a statistically significant difference between Abemaciclib-treated, Celecoxib-treated, and combination 2-treated cells compared to the combination 1-treated cells (*p* < 0.0001). As for Caco-2 cells, the data presented in (Fig. 3D) inferred that LC3 protein levels were significantly increased in all the treated groups compared to the control group (*p* < 0.0001).

#### Influence of the treatment protocol on the pro-apoptotic protein, Caspase-3, in the investigated CRC cells after 48 h of treatment

**Fig. 4:**
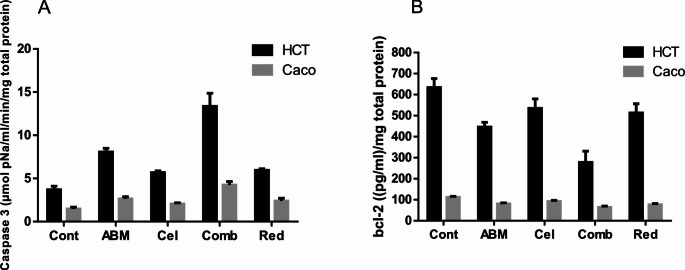
(**A**) Influence of the treatment protocol on Caspase-3 protein level in the investigated CRC cells after 48 h of treatment. a: *p* < 0.05 vs. control, b: *p* < 0.05 vs. Abemaciclib, c: *p* < 0.05 vs. combination 1, d: *p* < 0.05 vs. combination 2 where a, b, c, d denotes statistical significance of the treated groups versus each other as shown in the figure. (**B**) Influence of the treatment protocol on Bcl-2 protein level in the investigated CRC cells after 48 h of treatment. a: *p* < 0.05 vs. control, b: *p* < 0.05 vs. Abemaciclib, c: *p* < 0.05 vs. combination 1, d: *p* < 0.05 vs. combination 2 where a, b, c, d denotes statistical significance of the treated groups versus each other as shown in the figure

Our data in (Fig. 4A) depicted that Caspase-3 protein levels in HCT-116 cells were significantly increased in comparison to the control group by around 118%, 262%, and 61% in Abemaciclib-treated, combination 1-treated, and combination 2-treated cells, respectively (*p* < 0.0002, *p* < 0.0001, and *p* < 0.0259, respectively). Additionally, a statistically significant difference was found between Abemaciclib-treated, Celecoxib-treated, and combination 2-treated cells in comparison to the combination 1-treated cells (*p* < 0.0001). In Caco-2 cells; such levels were significantly elevated by about 79%, 187%, and 62% in Abemaciclib-treated, Combination 1-treated, and combination 2-treated cells respectively (*p* < 0.0269, *p* < 0.0120, and *p* < 0.0218, respectively) in relation to the control group. In addition, a statistically significant difference was found between all treatment groups compared to the combination 1-treated cells, as shown in (Fig. 4A).

#### Influence of the treatment protocol on the anti-apoptotic protein, Bcl-2, in the investigated CRC cells after 48 h of treatment

Pertaining to HCT-116 cells, as illustrated herein (Fig. 4B), Bcl-2 protein levels were decreased significantly by 30%, 56%, and 19% in comparison to the control in Abemaciclib-treated, combination 1-treated, and combination 2-treated cells, respectively (*p* < 0.0021, *p* < 0.0001, and *p* < 0.0378, respectively). The data given in (Fig. 4B) inferred that Abemaciclib, Celecoxib, and their combinations significantly decreased Bcl-2 levels in Caco-2 cells by 28%, 17%, 42%, and 31% in comparison to the control (*p* < 0.0001, *p* < 0.042, *p* < 0.0001, and *p* < 0.0001, respectively).

#### Influence of the treatment protocol on Cox-2 protein level in Caco-2 cells after 48 h of treatment

**Fig. 5:**
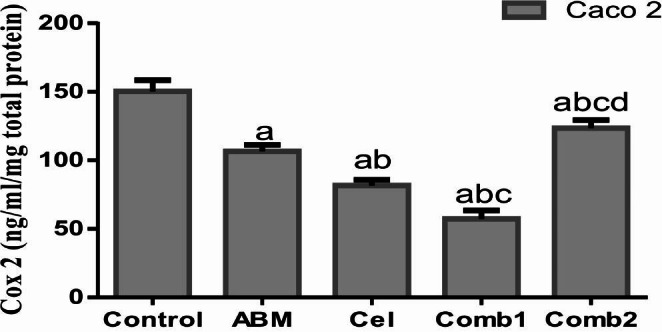
Influence of the treatment protocol on Cox-2 protein level in Caco-2 cells after 48 h of treatment. a: *p* < 0.05 vs. control, b: *p* < 0.05 vs. Abemaciclib, c: *p* < 0.05 vs. combination 1, d: *p* < 0.05 vs. combination 2 where a, b, c, d denotes statistical significance of the treated groups versus each other as shown in the figure

In Caco-2 cells, Cox-2 protein level was significantly reduced by 29%, 46%, 62%, and 18% when compared to the control group in Abemaciclib-treated, Celecoxib-treated, combination 1-treated, and combination 2-treated cells, respectively (*p* < 0.0001, *p* < 0.0001, *p* < 0.0001, and *p* < 0.0017, respectively), as illustrated in (Fig. 5). In addition, HCT-116 cells were found to be Cox-2 negative.

#### Influence of the treatment protocol on cell migration in the investigated CRC cells after 48 h of treatment

**Fig. 6:**
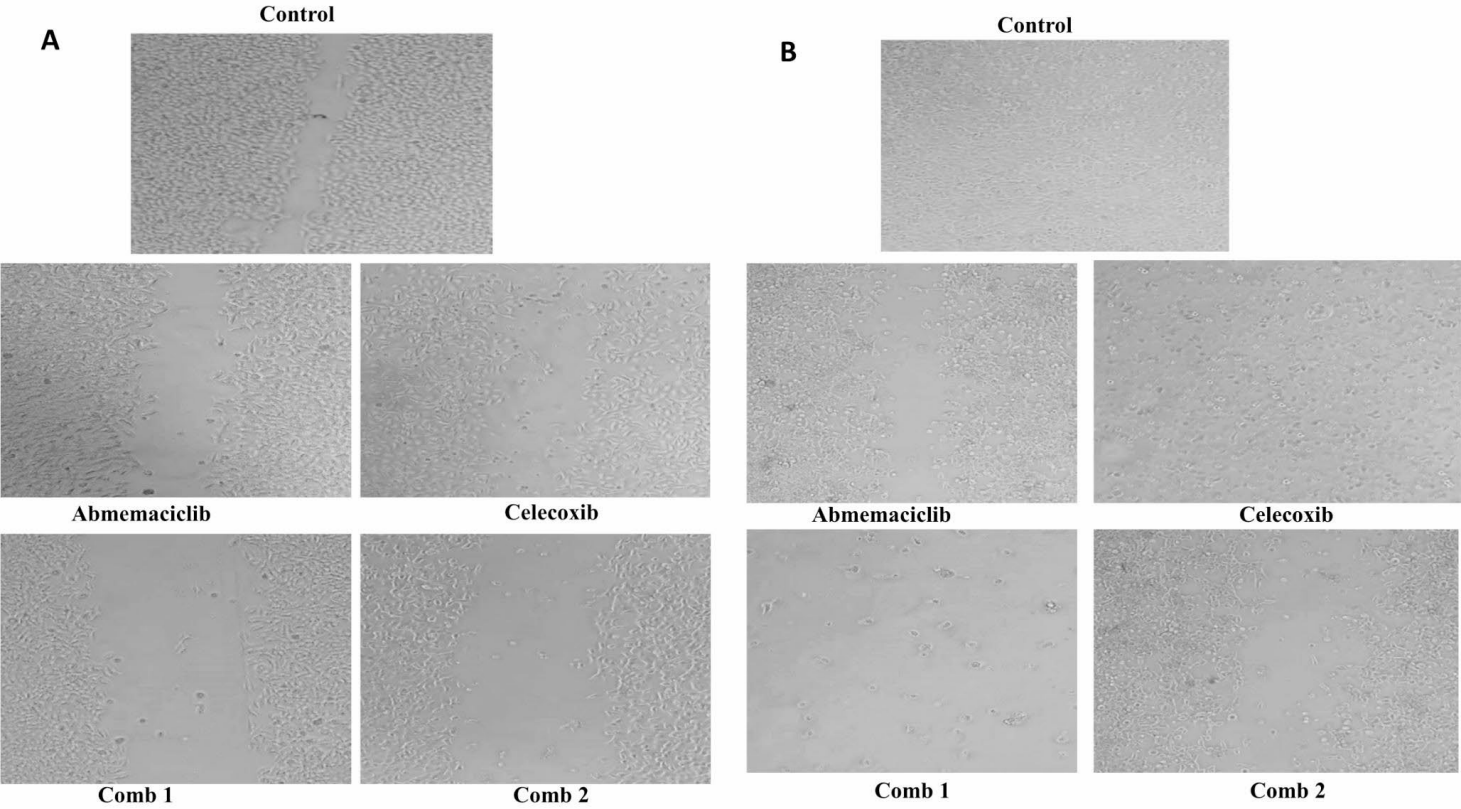
(**A**) Microscopic examination of cell migration for HCT-116 cell line at 24 h. (**B**) Microscopic examination of cell migration for HCT-116 cell line at 48 h

**Fig. 7:**
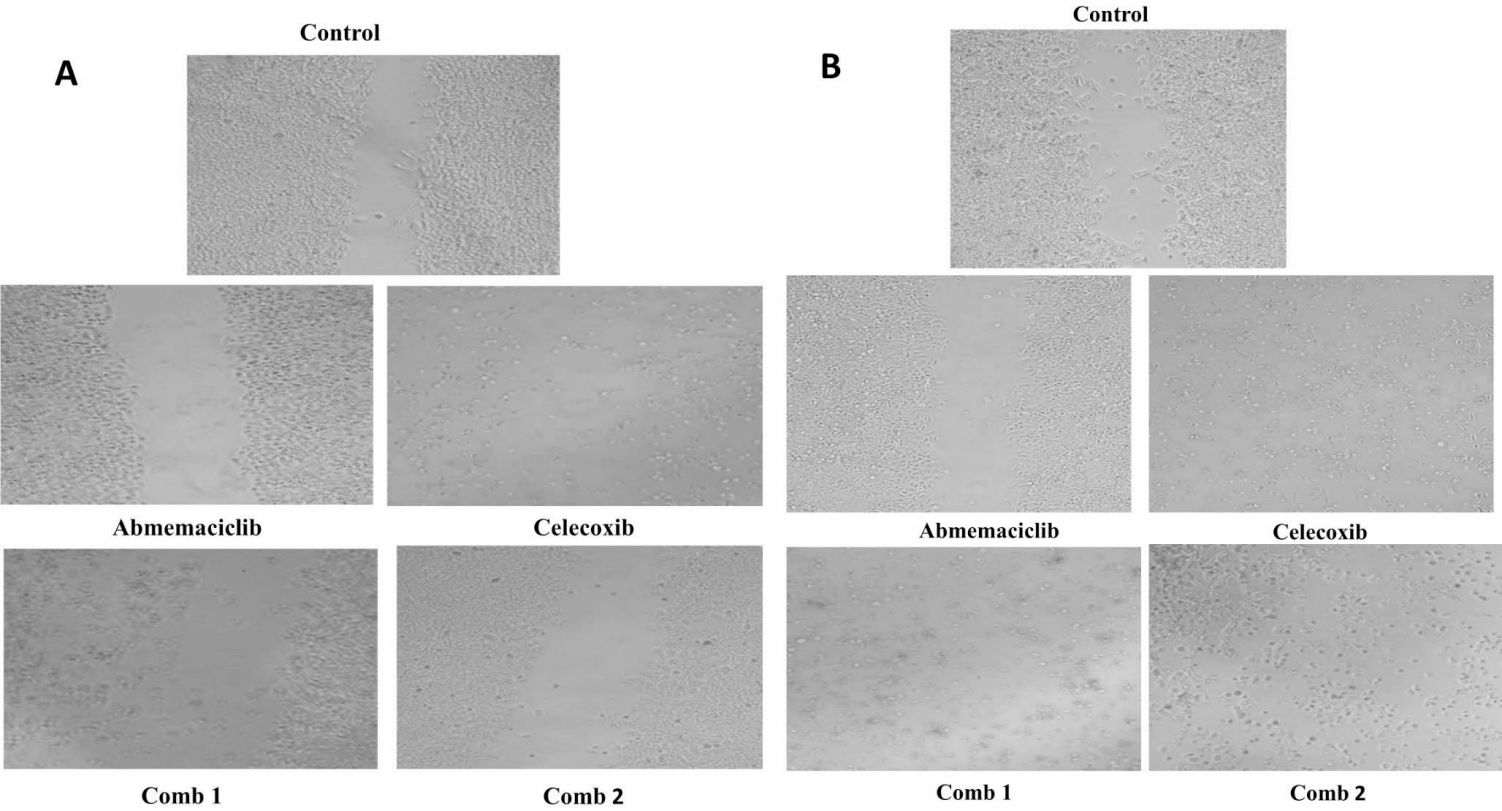
(**A**) Microscopic examination of cell migration for Caco-2 cell line at 24 h. (**B**) Microscopic examination of cell migration for Caco-2 cell line at 48 h

Our findings presented in (Fig. 6A) showed that the migratory cell % in HCT-116 cells for Abemaciclib, Celecoxib, combination 1, and combination 2 treated groups were about 17.5%, 12.5%, 1%, and 1.5%, respectively, in comparison to the control group after 24 h of treatment. Whereas, the percent was 5%, 0%, 0%, and 2%, respectively compared to the control after 48 h of treatment, as demonstrated in (Fig. 6B). In Caco-2 cells, the migratory cell % were about 20%, 23%, 0, and 8%, respectively, compared to the control after 24 h of treatment, as shown in (Fig. 7A). Whereas, it was 7%, 6%, 0%, and 0%, respectively, compared to the control group after 48 h of treatment, as given in (Fig. 7B).

## Discussion

Mounting data suggest that autophagy is deregulated in various cancer types, and its modulation holds great potential as a therapeutic approach. Autophagy has a double role in cancer, serving as a cytoprotective factor in some cases and a cytotoxic factor in others. Autophagic outcomes in various malignancies are context-dependent and not fully understood [15]. Knowing the role of autophagy in each cell type, as well as the underlying signaling pathways, is critical for controlling the impact on cell death and survival.

Numerous articles have highlighted the role of several cell cycle regulators in the modulation of the autophagic machinery. Herein, the CD4/6 inhibitor, Abemaciclib, was used. The rational beyond using Abemaciclib is its ability to induce cell cycle arrest and inhibit growth both *in-vivo* and *in-vitro* [16]. Non-steroidal anti-inflammatory drugs (NSAIDs) have been linked to autophagy in several studies, including hepatocellular carcinoma, glioblastoma, neuroblastoma, acute leukemia, lung adenocarcinoma, oral, breast, colon, bladder, and gastric cancers [17]. Our rational for utilizing Celecoxib is its ability to reduce growth, promote apoptosis, modulate tumor microenvironment, and reduce angiogenesis in colorectal cancer. Meanwhile, Celecoxib’s anticancer effects are enhanced by Cox-2-independent mechanisms [18].

In this sense, the present study aimed at investigating the influence of Abemaciclib, Celecoxib, and their combinations on the autophagic machinery in HCT-116 and Caco-2 colorectal cancer cell lines. Moreover, caspase-3 and Bcl-2 were also measured to unravel the crosstalk between autophagy and apoptosis in the investigated CRC cell lines.

The synergistic effect of Abemaciclib and Celecoxib was confirmed, as evidenced by the cell viability assay and the calculated combination index. This study inferred that the influence of the Abemaciclib/Celecoxib combination at their GI50s is better than that of each drug alone, which suggests the presence of a synergistic effect between them in both cell lines. However, the synergistic effect in HCT-116 cells is more pronounced than in Caco-2 cells. In addition to that, both drugs, either alone or in combination, elevated the levels of the autophagic markers suggestive of autophagy induction. Also, there is a decrease in the protein level of Bcl-2 and an increase in caspase-3 activity, suggesting apoptosis induction. Moreover, both drugs, either alone or in combination, decreased the migration ability of the cells in both cell lines, suggesting their inhibitory effect on cancer cell migration.

To start with, the treatment protocol increased the expression level of all tested autophagic markers. Supporting our findings, autophagy was found to be induced in response to CDK4/6 inhibitors in a previous study [19]. Abemaciclib triggered autophagy in multiple myeloma cell lines and showed cytocidal action with cytoplasmic vacuolization against myeloma cells dose dependently [20]. In Glioblastoma multiforme, the most aggressive brain tumor, Abemaciclib promoted autophagy activation [21]. The Abemaciclib-induced rise in LC3B in renal cell carcinoma cells supports the notion that Abemaciclib exposure causes an increase in autophagosomes. Also, Abemaciclib elevated Beclin-1 levels, suggesting an increase in autophagy [22].

As for celecoxib, it was found that Celecoxib increased LC3-II expression in hypoxic glioblastoma cells compared to normoxic glioblastoma cells, indicating that Celecoxib has an effect on autophagic cell death [23]. The same results were achieved in several cell lines including TNUB1 urothelial carcinoma cells, U87MG glioblastoma cells, MCF-7 breast cancer cells [17], PC3 prostate cancer cells [24]. It was documented that Celecoxib is an endoplasmic reticulum (ER) stress inducer and the cellular response to ER stress might be linked to autophagy induction after Celecoxib administration [24].

The data presented herein demonstrated that treatment of HCT-116 and Caco-2 cells with Abemaciclib, Celecoxib, and their combinations increased p62 expression, although it was expected to decrease upon autophagy induction, as it was reported earlier that activating autophagy reduces the expression of p62 [25]. Such an increase in p62 expression level in our study might be due to suppression of Wnt/b-catenin signaling when autophagy is induced, as it was previously reported that the Wnt/β-catenin signaling pathway serves as a negative regulator of autophagy in a plethora of studies [26].

The treatment protocol increased the Caspase-3 protein level, suggesting its ability to induce apoptosis. It was reported that CDK4/6 inhibitors cause apoptosis in cancer cells. Abemaciclib can promote Caspase-3 overexpression, early apoptosis, and G1 arrest in Pancreatic ductal adenocarcinoma (PDAC) cells via down-regulating p-Rb [27]. Abemaciclib suppresses cervical cancer cell growth and promoted apoptosis via the suppression of CDK4/6-Rb-E2F and mTOR pathways [10]. Abemaciclib suppressed breast tumors *in-vitro* and *in-vivo* by promoting apoptosis and senescence [28]. Abemaciclib enhanced apoptosis in PC3 cells by inhibiting the CDK4/6/Cyclin D complex, over-expression of Caspase-3, pro-apoptotic proteins (Bid, Bim), and cell cycle regulatory proteins (p53/p21/p27), as well as down-regulation of inhibitor of apoptosis 2, X-linked inhibitor of apoptosis protein, and Heat shock protein 60 [29]. Abemaciclib increased Caspase-3 in MDA-MB-231, MDA-MB-468, and MCF-10 A triple-negative breast cancer cells.

It was inferred that Celecoxib promoted apoptosis in colorectal cancer cell lines, which is how it exerts its anticarcinogenic action. Celecoxib inhibited the growth of colon tumors by inducing apoptosis through Cox-dependent and Cox-independent pathways [30]. Celecoxib also promotes apoptosis in breast cancer cell lines via the PGE2 pathway [12]. In cervical cancer cells, Celecoxib triggered apoptosis via the death receptor pathway [31]. In lymphoma, Celecoxib promoted apoptosis through an apoptosome-dependent mechanism [31]. Celecoxib dramatically elevated Caspase-3 in BJMC3879 mammary cancer cells, strongly supporting the activation of the intrinsic mitochondrial pathway [32]. It was reported that Celecoxib induced apoptosis via decreasing NF-kB activity in K562 cells [33], and glioblastoma cells [34]. Moreover, Celecoxib is implicated in Ca^2+^-sensitive proteases, endonucleases, and caspases activation. The concentration of Ca^2+^ affects the opening of mitochondrial permeability transition pores, which release cytochrome C [35]. As a result, Celecoxib-induced Ca^2+^ ATPase inhibition in the ER may provide a feasible link with Celecoxib’s apoptosis-inducing effect. Taken all together, Celecoxib targets a number of mechanisms in mediating apoptosis, including the activation of death receptor and mitochondrial pathways [36], as well as the decrease in the PI3K/AKT and β-catenin pathways [37].

It is worth mentioning here that Caspase-3 is at the crossroad between apoptosis and autophagy. Caspases are involved in apoptosis; however, previous research has found that Capase-3 is also involved in the autophagic process [38]. Caspase-3 promoted the export of autophagic vacuoles extracellularly in human apoptotic endothelial cells under nutritional deprivation by rerouting these autophagic vacuoles in the direction of the cell membrane [39]. The exportation of these big autophagic vacuoles may have a role in apoptotic volume reduction, which is a geometric predictor of cell breakdown into apoptotic bodies [40]. These findings point to Caspase-3 as a node involved in modulating the crosstalk between autophagic and apoptotic pathways [39].

What makes Abemaciclib a good choice, beside its potential to promote autophagy and apoptosis, is its ability to cause atypical cell death linked with the production of cytoplasmic vacuoles formed from the swollen lysosomes. Abemaciclib results in lysosomal acidification through H^+^ transport. The influence on V-ATPase seems to cause lysosomal enlargement because of H_2_O influx, as well as lysosomal malfunction and eventually cell death through a unique molecular pathway. It was reported that Abemaciclib caused such a cell death phenotype in prostate cancer cells, A549 non-small lung cancer carcinoma cells, and MDA-MB-231 triple negative breast cancer cells [41].

Herein, the antiapoptotic Bcl-2 protein was reduced by the treatment protocol. Mounting data support the notion that the Bcl-2 protein family seems to govern both apoptosis and autophagy. The *Bcl-2* gene produces the Bcl-2 protein that suppresses autophagy by attaching to Beclin-1’s BH3 domain and limiting its function. According to a previous study, the pro-survival Bcl-2 protein indirectly controls autophagy by blocking Bax and Bak [42]. Bcl-2 is linked to inhibition of apoptosis in CRC patients. Besides, the pivotal role of Bcl-2 in autophagy modulation has been examined in colon carcinoma, where the loss of the BH4 domain of the Bcl-2 protein did not influence tumorigenicity in the HT29 colon carcinoma cell line [43]. Abemaciclib lowered mRNA levels of the anti-apoptotic gene Bcl-2 in SW1736 and C643 thyroid carcinoma cells [44]. Celecoxib promoted apoptosis and autophagy in CRC cells, both of which are negatively regulated by Bcl-2/Bcl-XL [45].

Regarding the crosstalk between autophagy and apoptosis in CRC, it was discovered that autophagy and apoptosis have an intricate interrelation in CRC, involving a m**y**riad of cellular signal transduction cascades. The different interactions that could occur between autophagy and apoptosis in tumors are represented by synergistic, enhancing, and antagonistic effects [46]. The combination of Abemaciclib and Celecoxib appears to have either synergistic or enhancing effects.

Autophagy and epithelial-to-mesenchymal transition (EMT) have an intricate interrelation [47]. Autophagy induction has been found to suppress EMT in ovarian cancer cells [48]. Regarding our tested drugs, Celecoxib was reported to suppress EMT and cancer cell metastasis *in-vivo* in bladder cancer via microRNA-145 overexpression and transforming growth factor-receptor 2 and Smad family member 3 down-regulation [49]. Celecoxib suppresses EMT and invasiveness in HT-29 CRC cells [50]. Celecoxib inhibited EMT and lung cancer migration and invasion through silent mating type information regulation 2 homolog (SIRT-1) down-regulation [51]. Taken all together, Celecoxib inhibited EMT and cell mobility via blocking various transcriptional factors, cytoplasmic mediators, cell adhesion molecules, and surface receptors.

The influence of Abemaciclib on migration was assessed by a scratch wound healing assay, and it was found that Abemaciclib significantly inhibited cell migration in Caco-2 cells, consistent with the present findings [52]. Abemaciclib’s capacity to enhance active Rb protein levels and reduce NF-κB might be the cause of its anti-migratory effects. MMP-9, which has the ability to break down the extracellular matrix and facilitate cell migration to the bloodstream and/or lymph nodes, is directly activated by NF-κB. Additionally, snail protein, a transcriptional repressor for E-cadherin, is stabilized by NF-κB. Additionally, Rb protein acts as an E-cadherin transcriptional activator [53].

To conclude, the synergistic effect of Abemaciclib and Celecoxib was confirmed at the cellular level, as indicated by the cell viability assay, and calculated combination index. Both drugs and their combinations induced both autophagy and apoptosis. Notably, the effect of the combination was more pronounced than that of each drug alone. The atypical cell death mechanism of Abemaciclib will definitely add to the success of this drug combination to suppress proliferation and cell growth. Additionally, the combination inhibited the migration and invasion of cancer cells, as evidenced by the migration assay, plus both drugs were previously found to inhibit the PI3K/AKT/mTOR axis, hence inhibiting EMT and cancer metastasis. Future research is needed to explore the antitumor effects of these drugs and their combinations on different CRC cell lines, such as HT 29, that express different genotype profiles, and to assess the antitumor effects of these drugs and their combinations on other types of cancer cell lines, such as prostate cancer cells (e.g., PC3). Additionally, the anti-cancer potential of Abemaciclib, Celecoxib, and their combinations should be investigated *in-vivo* in various cancer types to verify the findings.

## Data Availability

‘The data generated and analyzed during the current study are available from the corresponding author upon request’.
